# The combined effect of high‐intensity intermittent training and vitamin D supplementation on glycemic control in overweight and obese adults

**DOI:** 10.14814/phy2.13684

**Published:** 2018-04-30

**Authors:** Hannah Margaret Lithgow, Geraint Florida‐James, Melanie Leggate

**Affiliations:** ^1^ Department of Sport and Exercise Science School of Applied Sciences Edinburgh Napier University Edinburgh United Kingdom

**Keywords:** glucose tolerance, high‐intensity intermittent training, vitamin D

## Abstract

High‐intensity intermittent training (HIIT) has been shown to reduce the risk of chronic conditions including the development of type 2 diabetes mellitus (T2DM). Independently, a low vitamin D status has also been linked to the prevalence of T2DM. The aim of this study was to investigate if there was a synergistic metabolic effect of HIIT and vitamin D supplementation on glycemic control. A total of 20 male and female participants (age, 34 ± 9 year; BMI, 31.4 ± 2.8 kg·m^−2^) completed 6 weeks HIIT, and were randomized to ingest 100 *μ*g·day^‐1^ of vitamin D_3_ or placebo. Response to an oral glucose tolerance test (OGTT) was determined at baseline and at 72 h postintervention. Glucose tolerance was improved as a result of the HIIT intervention, shown through a reduction in glucose and insulin concentrations during the OGTT, accompanied by a decrease in glucose (829 ± 110 to 786 ± 139 mmol·h^−1^·L^−1^; *P *=* *0.043) and insulin (8101 ± 4755–7024 ± 4489 mU·h^−1^·L^−1^; *P *=* *0.049) area under the curve (AUC). Supplementation increased 25‐hydroxyvitamin D_3_ concentration by 120% to a sufficiency status (*P *<* *0.001). However, the consumption of vitamin D_3_ seemed to attenuate the glucose response during an OGTT. Triglyceride content was lowered following the intervention (*P *=* *0.025). There was no effect of the intervention on insulin sensitivity (IS) indices: ISI_M__atsuda_ and HOMA‐IR. Our findings demonstrate that HIIT improves glucose tolerance in nondiabetic overweight and obese adults; however vitamin D_3_ supplementation did not proffer any additional positive effects on the measured indices of metabolic health.

## Introduction

Obesity and lack of regular exercise are reported to be the leading causes of chronic diseases (Booth et al. 2012) and global mortality (WHO, 2010), with exercise playing a major role in the prevention of type 2 diabetes mellitus (T2DM) (Pan et al. 1997; Boule et al. 2001; Knowler et al. 2002). The initial pathophysiological events in the development of T2DM are insulin resistance, glucose intolerance, and chronic hyperglycemia (Kahn 2003), suggesting interventions should address these underpinning metabolic syndromes. High‐intensity intermittent training (HIIT) and sprint interval training (SIT), involving repeated short bouts of high‐intensity exercise (above 85% of maximal aerobic capacity), have been reported to improve insulin sensitivity (IS) (Babraj et al. 2009) and glucose tolerance (Little et al. 2011, 2014).

In addition to the low exercise status in the UK (BHF, 2015), another contributor in the etiology of chronic diseases, such as T2DM, multiple sclerosis, and cardiovascular disease, is vitamin D deficiency (Holick 2004). The high northern latitude of the UK (50°–59°N) and the prevailing cloud cover and poor weather conditions does not facilitate sufficient vitamin D skin synthesis to increase or maintain a sufficient vitamin D status (Rhodes et al. 2010), and thus renders much of the nation vitamin D deficiency (Hypponen and Power 2007; Zgaga et al. 2011). The systemic concentration of 25‐hydroxyvitamin D_3_ (25[OH]D_3_) defines vitamin D status, with deficiency classified as a concentration of <20 ng·mL^−1^ and sufficiency as > 30 ng·mL^−1^ (Holick et al. 2011).

A low vitamin D status is often associated with metabolic disruptions, one of which is an increased risk of glucose intolerance (Chiu et al. 2004), with reports that obesity is inversely correlated with vitamin D status, attributable to the sequestering of fat‐soluble 25(OH)D by adipocytes (Wortsman et al. 2000). The inverse association reported between obesity and maintaining euglycemia, suggests that excess fat mass is a risk factor in the etiology of the disease (Must et al. 1999). However, there is conflicting evidence surrounding the efficacy of vitamin D_3_ supplementation alone to improve glucose tolerance, and little research investigating a combined effect of vitamin D_3_ supplementation and exercise training.

A recent study reported vitamin D insufficiency to be linked to reduced glucose tolerance compared to individuals with a sufficient status (Kobza et al. 2013). However, the effect of baseline vitamin D status was independent of the effect of resistance training on improvements in glucose tolerance. However, supplementation with vitamin D_3_ has been demonstrated to increase vitamin D concentration (Barker et al. 2013) and expression of the key receptor that mediates the transcriptional action of vitamin D, the vitamin D receptor (VDR), effectively (Ceglia et al. 2013). It has recently been reported that vitamin D supplementation alone as an intervention may not improve insulin sensitivity or glycemic control in an overweight and obese population (Mousa et al. 2017). Research suggests that the active vitamin D metabolite, 1,25(OH)_2_D_3_, acts directly through the VDR to stimulate insulin biosynthesis or suppression (Maestro et al. 2000, 2002; Tiosano et al. 2001), therefore suggesting scope for vitamin D to influence glycemic control through insulin bioavailability. Studies have also shown that mechanical stress, in the form of exercise, that is HIIT, can alter the expression and action of 1,25(OH)_2_D_3_ and the VDR (Makanae et al. 2015; Aly et al. 2016). This may create an interlinking pathway between exercise‐induced metabolic and cellular alterations and cellular vitamin D metabolism, in order to influence glucose tolerance. The purpose of this investigation was to examine the combined effects of exercise in for the form of HIIT and vitamin D_3_ supplementation on glycemic control in an overweight and obese adult population.

## Materials and Methods

### Ethical approval

The study was approved by the School of Applied Sciences Research Integrity Approvals Group at Edinburgh Napier University in July 2014 (reference number: FHLSS/1000). The study and procedures conformed to the standards set by the latest version of the Declaration of Helsinki or the version that was in place at the time of the experiments. Informed written and verbal consent was obtained from all participants.

### Participants

A total of 22 overweight and obese Caucasian males (*n* = 15) and females (*n* = 7) were recruited to participate in the study (age 19–45 year). All of the volunteers had a body mass index (BMI) between 27 and 35 kg·m^−2^ but were otherwise healthy and did not engage in more than two bouts of light to moderate intensity exercise per week. Participants were excluded if they were a smoker, used tanning beds, took supplements containing vitamin D, or were routinely taking anti‐inflammatory drugs, statins, or steroids. Data collection and sampling took place over months from October to June to account for seasonal variation in vitamin D status in northern latitudes (Close et al. 2013).

### Baseline testing

The first visit to the laboratory involved basic anthropometric measurements, a maximal exercise test and familiarization to the HIIT protocol. Height, body mass, waist and hip circumference, and supine blood pressure measurements were taken at baseline. Participants performed a peak oxygen uptake (*V̇*O_2peak_) test that involved a step increase of 25 W every 2 min on a stationary cycle ergometer (Velotron Pro, Racer Mate, USA), maintaining a pedaling cadence of 70 rpm until volitional exhaustion (Yoon et al. 2007). Expired air was continuously analyzed via an on‐line breath‐by‐breath gas analysis system (Cortex, MetaLyzer 3B, Germany), and heart rate (HR) was monitored throughout the test by a chest worn HR monitor (Polar, RS400, Finland). The test was terminated when the participant could not maintain a pedaling cadence of >55 rpm. Verbal encouragement was given throughout the test. The value used for *V̇*O_2peak_ corresponded to the highest value averaged over 30 sec during the test. Following a 30‐min rest, participants performed a familiarization trial of the HIIT protocol consisting of five 1 min intervals (see Training).

Five days after the first session, participants returned to the laboratory between 0700 and 0900 following a 10‐h overnight fast to complete a standard 75 g oral glucose tolerance test (OGTT) (WHO, 1980). Participants refrained from strenuous physical activity the day prior to the OGTT, and completed a 24‐h food diary, which they were asked to replicate in the day prior to the postintervention OGTT. A fasted blood sample was collected into K_3_ ethylenediaminetetraacetic acid (EDTA) (approximately 1.0 mg) and sodium fluoride/potassium oxalate vacutainers via a cannula inserted into the antecubital vein, then participants consumed 75 g glucose dissolved in 290 mL of water and 10 mL of lemon juice over a 5‐min period. Further blood samples were subsequently taken via the indwelling cannula every 30 min for a period of 2 h. The cannula was kept patent via regular flushing with 0.9% saline solution. Participants were required to remain in a seated position for the 2‐h period. Whole blood was centrifuged (Satorius Universal, 320R, Germany) within 30 min of collection at 1500 *g* for 15 min at 4°C, and the resulting plasma aliquoted into Eppendorfs and stored at −80°C for subsequent analysis.

### Training

The 6‐week supervised HIIT intervention was initiated within 1–3 days following the OGTT, and consisted of 3 sessions/week in the laboratory separated by 1–2 days. All sessions were performed on the same stationary cycle ergometer (Velotron Pro) as the *V̇*O_2peak_ test, starting with a 5‐min warm up and ending with a 5‐min cool down at a power output of 50 W. The protocol was modeled on recent studies (Little et al. 2011, 2014; Mancilla et al. 2014) consisting of 10 repetitions of 1 min intervals interspersed with 1 min active recovery at a power output of 50 W. The power output (W) of the high‐intensity intervals was assigned as a workload corresponding to 100% *V̇*O_2peak_ (sessions 1–6) to 110% (sessions 7–12), and ultimately 120% (sessions 13–18). Heart rate and rate of perceived exertion (RPE) (Borg 1998) were monitored throughout each session.

### Supplementation

Participants were randomized (1:1) to receive a 6‐week course of vitamin D_3_ supplements or placebo tablets (Placebo‐World, Powys, UK). The vitamin D_3_ supplement capsules (Solgar, New Jersey) contained a dose of 100 *μ*g (4000 IU) vitamin D_3_: the recommended upper intake limit set by the Institute of Medicine (IOM) (Ross et al. 2011). Distribution of the supplement and placebo remained blind to both the researcher and participants. The laboratory technicians distributed the supplements/tablets in Manilla envelopes and kept a record. Participants were asked to consume one tablet daily with breakfast each morning.

All baseline testing were repeated approximately 72 h after the last training session, with participants arriving to the lab following a 10‐h fast between 0700 and 0830, to ensure the postintervention OGTT was performed at the same time of the day as the preintervention OGTT. The session included the anthropometric measures, OGTT and *V̇*O_2peak_ test (following a 30‐min rest after the OGTT was completed).

### Blood sample analysis

All assays and analysis methods were performed in duplicate. Whole blood was analyzed for full blood cell count in vacutainers containing K_3_ EDTA using a hematology analyzer (Sysmex, XS 1000i). Plasma samples were thawed prior to analysis. Commercially available enzyme‐linked immunosorbent assay (ELISA) kits were used to determine plasma insulin (Mercodia, Uppsala, Sweden), 25(OH)D_3_ (IDS PLC, Tyne and Wear, UK), adiponectin (R&D Systems, Minneapolis, MN), and leptin concentrations (R&D Systems, Minneapolis, MN). Plasma glucose, total cholesterol, triglyceride, and high‐density lipid (HDL) cholesterol concentrations were analyzed using a bench top clinical chemistry analyzer (Randox, RX Monza, UK). Low‐density lipid (LDL) cholesterol concentration was estimated using the following equation: LDL cholesterol = Total cholesterol–(triglycerides/2.2)–HDL cholesterol (Friedewald et al. 1972). Insulin sensitivity and insulin resistance were calculated using the IS index (ISI_Matsuda_) proposed by Matsuda and DeFronzo (Matsuda and DeFronzo 1999), and the homeostatic model assessment of insulin resistance (HOMA‐IR) (Matthews et al. 1985).

### Statistical analysis

Data are presented as means ± SD. All statistical tests were performed using SPSS 23.0 statistical software (IBM Analytics, New York). Significance was accepted at *P < *0.05. Data were checked for normal distribution using the Shapiro–Wilk test. The baseline participant characteristics were checked for statistical difference between the two groups: placebo and vitamin D supplement group using independent samples *t*‐tests. All data, except the OGTT insulin data, are normally distributed and parametric tests were performed. The OGTT insulin data were log transformed prior to statistical analysis. A two‐way repeated measures analysis of variance (ANOVA) was used to analyze the systemic insulin and glucose response to the OGTT, separately, determining the main effect of time (pre‐ to postintervention OGTT trials), and condition (vitamin D_3_ supplementation and placebo)‐by‐time interactions. The dependent variable was condition, and the independent variable was glucose or insulin concentration across the five time points (0, 30, 60, 90, and 120 min) during the OGTT's (repeated measures) from pre‐ to postintervention.

A two‐way (2 × 2) ANOVA was used to determine the main effect of time (pre‐ to postintervention), and condition (vitamin D_3_ supplementation and placebo)‐by‐time interactions for all measured dependent variables pre‐ and postintervention: vitamin D concentration, adiponectin concentration, Leptin concentration, cholesterol concentration, triglyceride concentration, HDL and LDL cholesterol concentrations, fasting plasma glucose and insulin concentrations, IS_matsuda_, and HOMA‐IR.

## Results

Twenty participants completed the study, with two participants withdrawing due to alternative time commitments. Adherence to supplement and placebo consumption was self‐reported by participants and was reported as 100% compliance. Adherence to the HIIT sessions was reported as 99.4% compliance.

### Anthropometric and performance measurements

Baseline characteristics and postintervention measures of all participants (*n* = 20), the vitamin D (*n* = 10) group and the placebo group (*n* = 10), are shown in Table 1. There were no significant differences between groups at baseline. Both waist and hip circumference were reduced (*P *=* *0.019; *P *=* *0.002, respectively) as a result of the HIIT program; this was accompanied by a decline in the systolic blood pressure by 6 mmHg after the 6 weeks (*P *=* *0.004); however, there was no influence of vitamin D supplementation on these outcomes (Table 1). *V̇*O_2peak_ was increased after 6 weeks of HIIT in both absolute terms and relative to body mass by 12% (*P *<* *0.001). Similarly, absolute and relative peak power output increased from pre‐ to postintervention by 17% (*P *<* *0.001). Vitamin D supplementation did not influence the performance outcomes.

**Table 1:** Anthropometric, *V*O_2peak_ test, and metabolic measurements at baseline and 72‐h postintervention

	All		Placebo		Vitamin D		Overall effect of time (pre to post)	Condition (vitamin D supplement vs. placebo)‐by‐time (pre to post) interaction
	Preintervention	Postintervention	Preintervention	Postintervention	Preintervention	Postintervention	*P* Value	*P* Value
*N* (male:female)	20 (14:6)		10 (6:4)		10 (8:2)			
Age (year)	34 ± 9		34 ± 10		34 ± 9			
Height (m)	1.75 ± 0.10		1.70 ± 0.10		1.80 ± 0.10			
Body mass (kg)	96.6 ± 13.7	96.3 ± 12.7	97.0 ± 15.6	96.3 ± 13.8	96.2 ± 12.2	96.3 ± 12.2	0.688	0.524
BMI (kg·m^−2^)	31.4 ± 2.8	31.3 ± 2.5	32.3 ± 3.1	32.1 ± 2.6	30.5 ± 2.2	30.5 ± 2.2	0.610	0.540
Waist circumference (cm)	99.4 ± 10.3	96.9 ± 9.7 a	100.6 ± 12.6	98.3 ± 11.3	98.2 ± 7.9	95.5 ± 8.2	0.019	0.799
Hip circumference (cm)	111.0 ± 5.9	109.4 ± 6.8 a	112.9 ± 5.8	111.5 ± 7.3	109.1 ± 5.6	107.4 ± 5.8	0.002	0.670
Waist‐to‐hip ratio	0.90 ± 0.09	0.89 ± 0.07	0.89 ± 0.11	0.88 ± 0.08	0.90 ± 0.07	0.89 ± 0.06	0.233	0.937
Resting HR (bmp)	70 ± 10	68 ± 8	71 ± 10	70 ± 7	68 ± 9	66 ± 8	0.398	0.743
Systolic blood pressure (mmHg)	131 ± 10	124 ± 11 a	129 ± 8	124 ± 13	134 ± 12	123 ± 9	0.004	0.410
Diastolic blood pressure (mmHg)	80 ± 9	79 ± 7	80 ± 9	78 ± 9	81 ± 9	80 ± 4	0.477	0.835
Absolute *V̇*O_2peak_ (L·min^−1^)	2.71 ± 0.64	3.01 ± 0.67 a	2.61 ± 0.79	2.86 ± 0.81	2.82 ± 0.42	3.20 ± 0.44	0.000	0.174
Relative V̇O_2peak_ (mL·kg^−1^·min^−1^)	27.7 ± 4.8	30.9 ± 5.4 a	26.7 ± 5.7	29.4 ± 6.3	28.8 ± 3.6	32.6 ± 3.8	0.000	0.273
Absolute peak power output (W)	215 ± 45	250 ± 48 a	206 ± 45	237 ± 50	226 ± 32	266 ± 44	0.000	0.328
Relative peak power output (W·kg^−1^)	2.21 ± 0.40	2.58 ± 0.39 a	2.11 ± 0.39	2.45 ± 0.35	2.32 ± 0.39	2.71 ± 0.41	0.000	0.680

Data are presented as means ± SD.

a Denotes a significant difference from preintervention; *P *<* *0.05.

### Vitamin D concentration

**Figure 1:**
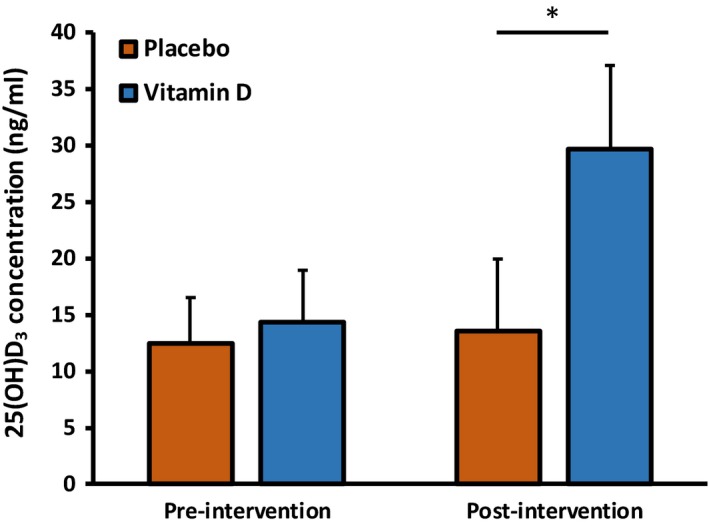
Plasma 25(OH)D_3_ concentrations at baseline and postintervention for the placebo group and the vitamin D group. *Denotes a significant condition (vitamin D supplement vs. placebo)‐by‐time (pre to post) interaction effect; *P *<* *0.05.

There was an overall effect of the intervention on plasma 25(OH)D_3_ concentration (*P *<* *0.001; Figure 1), with a clear effect of vitamin D supplementation, whereby the plasma 25(OH)D_3_ concentration of the vitamin D supplemented group increased from 14.4 ± 4.6 ng·mL^−1^ at baseline to 29.7 ± 6.9 ng·mL^−1^ (*P *<* *0.001). The placebo group exhibited no change in plasma 25(OH)D_3_ concentration (12.5 ± 4.0–13.6 ± 5.7 ng·mL^−1^) showing the comparative effect of a lack of vitamin D supplementation.

### Insulin sensitivity and OGTT outcomes

**Figure 2:**
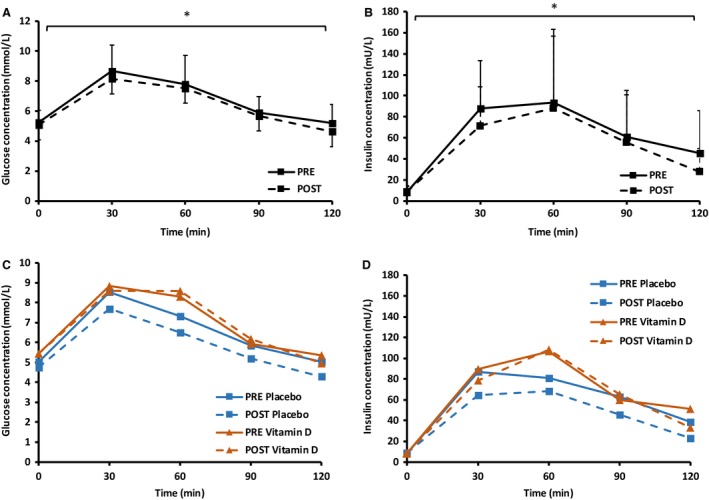
Response to a 75 g OGTT at baseline and 72 h postintervention for all participants: glucose (A) and insulin (B) concentrations, and split for the placebo and vitamin D supplement group: glucose (C) and insulin (D) concentrations. *Denotes a significant difference in the oral glucose tolerance test between pre‐ and postinterventions; *P *<* *0.05.

Glucose tolerance was improved through the reduction in circulating plasma glucose concentrations (*P *=* *0.027, Fig. 2A) and insulin concentrations (*P *=* *0.017, Fig. 2B) during the 120‐min OGTT performed at baseline and postintervention, with an effect of supplementation on the response (*P *=* *0.038). There was a reduction in both glucose AUC (*P *=* *0.043) and insulin AUC (*P *=* *0.049) as an overall effect of the intervention, with an effect of supplementation observed on glucose AUC (*P *=* *0.035) whereby vitamin D supplementation attenuated the improvement in glucose AUC (Table 2). Insulin sensitivity did not change significantly from pre‐ to postintervention, as assessed by ISI_Matsuda_ (*P *=* *0.087) and HOMA‐IR (*P *=* *0.676).

**Table 2:** Blood parameters at baseline and 72‐h postintervention

	All (*n* = 20)		Placebo group (*n* = 10)		Vitamin D group (*n* = 10)		Overall effect of time (pre to post)	Condition (vitamin D supplement vs. placebo)‐by‐time (pre to post) interaction
	Preintervention	Postintervention	Preintervention	Postintervention	Preintervention	Postintervention	*P* Value	*P* Value
Fasting glucose (mmol·L^−1^)	5.23 ± 0.83	5.09 ± 0.96	5.02 ± 0.83	4.74 ± 0.60	5.43 ± 0.94	5.44 ± 1.14	0.410	0.393
Fasting insulin (mU·L^−1^)	8.45 ± 5.30	8.88 ± 5.67	8.37 ± 4.55	8.84 ± 5.52	8.53 ± 6.24	8.91 ± 6.11	0.510	0.946
Glucose AUC (mmol·h^−1^·L^−1^)	829 ± 110	786 ± 139 a	802 ± 108	717 ± 114 a	855 ± 110	857 ± 130	0.043	0.035
Insulin AUC (mU·h^−1^·L^−1^)	8101 ± 4755	7024 ± 4489 a	7629 ± 5059	5846 ± 4175	8572 ± 4653	8201 ± 4694	0.049	0.099
ISI_Matsuda_	8.15 ± 7.73	12.35 ± 16.31	8.55 ± 7.74	16.49 ± 20.67	7.76 ± 8.12	8.21 ± 9.79	0.087	0.124
HOMA‐IR	2.06 ± 1.53	2.14 ± 1.64	1.90 ± 1.04	1.91 ± 1.23	2.23 ± 1.95	2.37 ± 2.01	0.676	0.737
Adiponectin (*μ*g·mL^−1^)	8.4 ± 4.5	7.1 ± 4.0 a	9.8 ± 5.8	8.3 ± 4.8	6.9 ± 2.3	6.0 ± 2.8	0.004	0.484
Leptin (ng·mL^−1^)	20.3 ± 16.4	20.0 ± 14.9	24.8 ± 19.7	23.7 ± 17.8	15.8 ± 11.5	16.2 ± 10.9	0.799	0.571
Cholesterol (mmol·L^−1^)	4.89 ± 0.91	4.93 ± 0.75	4.92 ± 1.14	5.11 ± 0.98	4.86 ± 0.68	4.75 ± 0.40	0.809	0.329
Triglycerides (mmol·L^−1^)	1.93 ± 1.06	1.72 ± 1.04 a	2.16 ± 1.31	1.87 ± 1.32	1.71 ± 0.73	1.57 ± 0.71	0.025	0.443
HDL‐cholesterol (mmol·L^−1^)	1.21 ± 0.28	1.22 ± 0.33	1.28 ± 0.30	1.32 ± 0.41	1.15 ± 0.25	1.13 ± 0.20	0.749	0.390
LDL‐cholesterol (mmol·L^−1^)	2.80 ± 0.97	2.92 ± 0.80	2.66 ± 1.21	2.94 ± 1.06	2.93 ± 0.70	2.91 ± 0.48	0.384	0.999

Data are presented as means ± SD.

a Denotes a significant difference from preintervention; *P *<* *0.05.

### Other metabolic markers

There was a decrease in adiponectin concentration as a result of the 6‐week intervention (*P *=* *0.004); however, there was no effect of supplementation observed (Table 2). There were no differences observed in leptin concentration in response to the intervention (Table 2). Circulating triglyceride content was lowered as a result of the intervention, with a main effect of time observed (*P *=* *0.025), although there was no effect of supplementation on triglyceride levels (*P *=* *0.443; Table 2). No differences were observed from pre‐ to postintervention for total cholesterol, HDL or LDL cholesterol (Table 2), or any of the hematological parameters that were measured (Table 3).

**Table 3:** Hematological parameters at baseline and 72‐h postintervention

	All (*n* = 20)		Placebo group (*n* = 10)		Vitamin D group (*n* = 10)		Overall effect of time (pre to post)	Condition (vitamin D supplement vs. placebo)‐by‐time (pre to post) interaction
	Preintervention	Postintervention	Preintervention	Postintervention	Preintervention	Postintervention	*P* value	*P* value
WBC (10^3^·*μ*L)	6.15 ± 1.56	6.02 ± 1.46	6.03 ± 1.50	5.84 ± 1.47	6.27 ± 1.69	6.20 ± 1.51	0.643	0.166
RBC (10^3^·*μ*L)	4.88 ± 0.31	4.85 ± 0.54	4.79 ± 0.39	4.86 ± 0.71	4.97 ± 0.17	4.83 ± 0.33	0.604	0.808
Hb (g·dL)	14.4 ± 0.9	14.4 ± 1.6	14.2 ± 0.9	14.4 ± 2.0	14.7 ± 0.9	14.4 ± 1.2	0.870	0.255
Hct (%)	42.6 ± 2.3	42.0 ± 5.6	42.1 ± 2.7	43.0 ± 5.9	43.2 ± 1.8	41.1 ± 5.4	0.527	0.127
PLT (10^3^·*μ*L)	254 ± 60	256 ± 93	259 ± 64	255 ± 76	250 ± 57	256 ± 112	0.924	0.616
Neutrophils (10^9^·L)	3.50 ± 1.30	3.27 ± 0.95	3.54 ± 1.43	3.31 ± 0.94	3.47 ± 1.25	3.23 ± 1.00	0.308	0.735
Lymphocytes (10^9^·L)	1.91 ± 0.45	2.00 ± 0.52	1.82 ± 0.41	1.79 ± 0.46	2.00 ± 0.50	2.21 ± 0.51	0.115	0.030
Monocytes (10^9^·L)	0.48 ± 0.10	0.53 ± 0.18	0.53 ± 0.12	0.53 ± 0.21	0.44 ± 0.06	0.52 ± 0.15	0.139	0.160
Eosinophils (10^9^·L)	0.22** **±** **0.18	0.19 ± 0.12	0.21 ± 0.10	0.18 ± 0.10	0.23 ± 0.25	0.20 ± 0.15	0.264	0.959
Basophiles (10^9^·L)	0.03 ± 0.01	0.03 ± 0.01	0.03 ± 0.01	0.03 ± 0.01	0.03 ± 0.01	0.03 ± 0.01	0.256	0.366

Data are presented as means ± SD. WBC, white blood cell count; RBC, red blood cell count; Hb, hemoglobin; Hct, hematocrit; PLT, platelets.

## Discussion

In the present study, the findings indicate that 6 weeks of HIIT improves glucose tolerance, shown through a reduction in systemic glucose concentration and the concentration of insulin required to mediate euglycemia (AUC data). There appears to be an attenuation of the HIIT‐induced improvement in glucose AUC with vitamin D supplementation. Contrary to the hypothesis, the absence of vitamin D_3_ supplementation induced a greater reduction in circulating glucose concentration during the OGTT compared to vitamin D consumption, which successfully elevated vitamin D status to sufficiency (>30 ng·mL^−1^). The intervention did not affect indices of IS (ISI_Matsuda_ and HOMA‐IR); however, this may be attributed to the relatively small sample size, underpowered, and the interindividual variability in the insulin data. The HIIT protocol adopted in the current investigation and previously used in other studies (Little et al. 2011, 2014; Mancilla et al. 2014) was effective at improving aerobic capacity and physical performance, while other HIIT‐induced significant physiological findings were a reduction in adiponectin concentration and triglyceride content.

Glycemic control is an important metabolic aspect in the prevention of T2DM and is identified as a risk factor in the development of diabetic complications (Woerle et al. 2007). Although no change was found in the ISI_Matsuda_ or HOMA‐IR, there was a reduction in postprandial glucose concentration accompanied by a decrease in plasma insulin concentration, suggesting systemic glycemic regulation is improved. Similar to our results, improvements in glycemic control have been reported in overweight and obese adults after just 2 weeks (6 sessions) of HIIT (Whyte et al. 2010), with 12 weeks inducing considerable reductions in insulin resistance in overweight adults with T2DM (Mitranun et al. 2014) and without T2DM (Racil et al. 2013). The latter studies differ to our current investigation in that the HIIT program involved either shorter (30 sec) maximal sprints (Whyte et al. 2010; Racil et al. 2013) or the training induced a reduction in BMI (Mitranun et al. 2014). Although in the current study the authors observed a decrease in waist and hip circumference, and thus, a beneficial change in body fat distribution or body composition, there was no observed change in body mass. It should also be considered that diet was not controlled or monitored during the 6 weeks intervention, although participants were requested to maintain their habitual diet and activity.

Furthermore, fasting plasma insulin and glucose remained unchanged; therefore, a change in HOMA‐IR may be unlikely despite a change in mean plasma glucose and insulin concentration during the OGTT. However, ISI_Matsuda_ is calculated from both fasting values and changing values during the OGTT; therefore should be possible to detect changes in IS, however a greater sample size may be required. Although, a HIIT‐induced reduction in HOMA‐IR was observed following 6 weeks of a similar exercise protocol, and was retained after 3 weeks cessation of training (Phillips et al. 2017). The current study population was nondiabetic and had normal fasting glucose and insulin concentrations; therefore, the intent of the intervention was not to alter fasting values but to improve glucose tolerance and thus reduce the requirement of insulin to control systemic glucose.

An interesting finding of the study was an attenuation of the HIIT‐induced improvement in glucose concentration during the OGTT, improving the body's euglycemic environment. This was contrary to the hypothesis, and may be attributed to the interindividual variability measured during glucose tolerance tests combined with small sample sizes, as reported in the HERITAGE Family Study (Boule et al. 2005). The inconsistent response by individual participants has also been reported by small training studies utilizing the same protocol but reporting different effects on insulin sensitivity (Metcalfe et al. 2012, 2016).

The timing of the postintervention blood sampling should be considered. Methodology differs between studies with regard to analysis of IS: OGTT or the gold standard hyperinsulinemic euglycemic clamp, as does timing of the test: 15–72 h posttraining. In the current study, the postintervention OGTT was performed 72 h after the cessation of the intervention to prevent any residual effects of the last HIIT session on blood markers. Studies that performed the OGTT 24 h after the final HIIT bout may reflect the combined impact of acute and chronic exercise training (Rogers 1989). The difference between the response to a 75 g OGTT at 24 h and 72 h post‐2 weeks of training has been demonstrated by Whyte et al. (2010), whereby they reported an increase in the ISI_Matsuda_ after 24 h but the impact was lost at 72 h. Therefore, the lack of effect on ISI_Matsuda_ and HOMA‐IR in the current study at 72 h postintervention could be attributed to the loss of the acute molecular response. However, an increase in IS expressed by the Cederholm Index, has been reported (in men only) at the 72 h time point following a 6‐week HIIT intervention (Metcalfe et al. 2012). Although, the Cederholm Index is not as commonly reported in epidemiological studies as the Matsuda Index, as the correlation coefficient with the gold standard hyperinsulinemic‐euglycemic clamp (HEC) is relatively lower (Piche et al. 2007), and therefore is not as reliable a measure to estimate IS.

Our findings revealed that 17 out of the 20 participants were classified as vitamin D deficient (<20 ng·mL^−1^) at baseline; however, the remaining 3 participants had concentrations of 20.6, 21.0, and 21.2 ng·mL^−1^ and thus were only marginally classified as insufficient. The findings support the current reports of widespread vitamin D deficiency in the UK (Hypponen and Power 2007; Zgaga et al. 2011), linked to low ultraviolet (UV) exposure (Kelly et al. 2015) and that supplementation (with 100 *μ*g 25(OH)D_3_) can raise vitamin D_3_ concentration to a sufficient status (>30 ng·mL^−1^). Studies investigating the effects of 25(OH)D_3_ supplementation have utilized a range of daily and weekly doses. The relatively high dose used in the current study was selected based on the susceptibility of overweight adults in Scotland to be generally vitamin D deficient (Bolland et al. 2007).

It has been established that a dose‐dependent relationship exists for vitamin D_3_ supplementation (Gallagher et al. 2012; Ekwaru et al. 2014), with baseline concentration influencing the response to supplementation. The current study observed relatively low baseline 25(OH)D_3_ concentrations and thus the concentrations postintervention were not elevated to the same extent as studies prescribing higher doses for shorter periods. Therefore, it may be that an increase in 25(OH)D_3_ alone is less relevant than the absolute concentration, with metabolic benefits attributed to a high sufficiency status. It is possible that no additional benefit of vitamin D_3_ supplementation in conjunction with the HIIT program was observed as participants were deficient or insufficient for the majority of the 6‐week intervention, due to a 4‐ to 6‐week lag time with supplementation before normalization of systemic 25(OH)D_3_ concentrations (Vieth 1999). Future studies investigating the combined effect of vitamin D and exercise training should supplement participants prior to commencement of the training intervention, to address this confounding factor.

Two key hormones expressed in human adipose cells, adiponectin and leptin, are reported to be linked to glucose regulation (Lihn et al. 2005), with both HIIT and vitamin D_3_ supplementation independently shown to influence concentrations of the proteins (Trapp et al. 2008; Leggate et al. 2012; Belenchia et al. 2013; Breslavsky et al. 2013; Racil et al. 2013; Ghavamzadeh et al. 2014). Adiponectin has been suggested to favor the uptake of glucose into skeletal muscle and thus is regarded as a predictor for reducing hyperglycemia (Punthakee et al. 2006). Therefore, it would appear that higher circulating adiponectin would be favorable, which has independently been reported following 12 weeks HIIT (Racil et al. 2013) and 12 months vitamin D_3_ supplementation (Breslavsky et al. 2013). In contrast to these reports, and in line with our result, adiponectin has been reported to decrease following HIIT (Leggate et al. 2012), and after a course of vitamin D_3_ supplementation (Belenchia et al. 2013). Since adiponectin is an adipose tissue derived protein hormone, it has been suggested that fat mass variations override the effect of exercise on systemic adiponectin (Christiansen et al. 2010; Gastebois et al. 2016), and thus, the observed decrease in adiponectin may be due to the reduction in waist and hip circumference. Furthermore, exercise training alone can reverse adiponectin resistance at the skeletal muscle level, thereby reducing the requirement for bioavailable adiponectin (Van Berendoncks et al. 2010, 2011). Additionally, leptin is often associated with adiponectin, with an elevation in the adiponectin‐to‐leptin ratio reported to correlate with a rise in fasting glucose (Finucane et al. 2009). Changes in leptin have however been attributed to alterations in body mass, although this was not observed in the current study. Similarly, alterations in the lipid profile have been associated with improvements in fat metabolism and body mass (Racil et al. 2013), which may supersede the effect of HIIT on fat metabolism.

## Conclusions

To our knowledge, no prior study has investigated the combined effects of HIIT and vitamin D_3_ supplementation on physical and metabolic parameters that influence glycemic control. The key finding of the current study is that 6 weeks of regular HIIT (3 sessions/week) improved glucose tolerance in a nondiabetic overweight and obese population, accompanied by a reduction in adiponectin concentration and systemic triglyceride content. Vitamin D_3_ supplementation increased circulating 25(OH)D_3_ concentration to a sufficient status; however, it unexpectedly attenuated the HIIT‐induced improvement in glucose AUC. Further studies are required to investigate the mechanisms involved in altering glucose tolerance and IS following chronic adaptations to HIIT.

## Conflict of Interest

The authors declare that they have no conflict of interest.
